# Influenza Hemagglutinin and Neuraminidase: Yin–Yang Proteins Coevolving to Thwart Immunity

**DOI:** 10.3390/v11040346

**Published:** 2019-04-16

**Authors:** Ivan Kosik, Jonathan W. Yewdell

**Affiliations:** Laboratory of Viral Diseases, NIAID, NIH, Bethesda, MD 20892, USA; ivan.kosik@nih.gov

**Keywords:** Influenza A virus, hemagglutinin, neuraminidase, viral evolution, antigenic drift

## Abstract

Influenza A virions possess two surface glycoproteins—the hemagglutinin (HA) and neuraminidase (NA)—which exert opposite functions. HA attaches virions to cells by binding to terminal sialic acid residues on glycoproteins/glycolipids to initiate the infectious cycle, while NA cleaves terminal sialic acids, releasing virions to complete the infectious cycle. Antibodies specific for HA or NA can protect experimental animals from IAV pathogenesis and drive antigenic variation in their target epitopes that impairs vaccine effectiveness in humans. Here, we review progress in understanding HA/NA co-evolution as each acquires epistatic mutations to restore viral fitness to mutants selected in the other protein by host innate or adaptive immune pressure. We also discuss recent exciting findings that antibodies to HA can function in vivo by blocking NA enzyme activity to prevent nascent virion release and enhance Fc receptor-based activation of innate immune cells.

## 1. Introduction

Year in and year out, influenza A virus (IAV) imposes an enormous economic and health burden, with the potential to cause periodic catastrophic pandemics. IAV is an enveloped negative stranded RNA virus with a segmented genome belonging to the Orthomyxoviridae family. Eight gene segments code for 10 structural and at least 9 nonstructural/regulatory proteins [1,2,3]. PB1, PB2, PA, NP, M1, NS1, and NEP are present inside the lipid envelope, while M2, hemagglutinin (HA), and neuraminidase (NA) are embedded in the envelope and available for antibody (Ab) binding.

Inactivated IAV vaccines induce antibody (Ab) responses to the HA, although it is now appreciated that NA might be an important target as well [4]. The high mutational tolerance [5] of these surface glycoproteins, both structurally and functionally compared to other IAV gene products [6], facilitates their “antigenic drift”—immune escape from Ab responses based on mutant selection [7]. Glycoprotein change is enhanced by the segmented nature of IAV genome, which facilitates intergenic epistasis through rapid recombination of mutant genes. Such recombination occurs rapidly in vivo [8,9,10,11,12] and enables antigenic shift, a process that introduces novel HA and NA genes from the enormous animal reservoir into the human IAV virome [9].

Eighteen hemagglutinin and 11 neuraminidase subtypes are known to exist in nature. All but H17N10 and H18N11 subtypes, found to date in Peruvian bats [13,14], circulate in wild aquatic birds, which is by far the largest of the known natural IAV reservoirs, which also include humans, swine, horses, dogs, and seals. Based on sequencing data available in GenBank, out of the 144 possible HA-NA combinations in non-bat IAVs, over 120 combinations have been documented in nature [15,16]. While many combinations are possible, far fewer are prevalent in nature, consistent with the co-evolution of HA and NA subtypes. Here we review the functional, genetic, and immunological interactions of the HA and NA.

## 2. HA Attaches, NA Releases

HA is a homotrimer whose globular domain contains a receptor binding site (RBS) specific for sialic acid (SA), which terminates many host glycans. The RBS is surrounded by antigenic sites recognized by the most potent virus neutralizing Abs. HA initiates infection by attaching virus to SA and possibly other receptors on the target cell surface [17,18]. Attachment is a complex process influenced by multiple parameters. The avidity of a single HA trimer for SA is low, with mM to high μM Kd values. However, multivalent binding of multiple HA trimers on the virion results in 10^4^- to 10^6^-fold increase in avidity [19,20,21,22], making attachment essentially irreversible in the absence of mitigating factors (e.g., NA or attachment blocking Abs). The effect of virion morphology on binding is potentially important, as freshly isolated viruses are typically filamentous, becoming more spherical (~100 nm diameter) during adaptation to cultured cells or eggs [23,24]. While intuition suggests that filaments should bind cells better than spheres, the available data suggest otherwise [25,26].

The specificity of HA for various types of SA linkage is a major contributor to their host and organ tropism. HA from human isolates generally prefer α2,6-linked SAs, while avian lineage HAs favor α2,3 linkages [25,27]. α2,6-linked SA glycan preference appears to dictate a requirement for fibronectin to initiate infection post attachment [28], pointing to unappreciated subtleties in how HA-mediated attachment leads to productive infection. The α2,6-α2,3-linked human–avian dichotomy is a gross oversimplification of HA specificity, and there is evidence that HA specificity can vary successively among human isolates. While an α2,3-linked SAs preference is associated with enhanced pathogenicity, it can also impair replication and aerosolization [29,30,31]. On the other hand, there are reports that SA-binding specificity has no apparent effect on IAV transmissibility or pathogenicity [32,33,34], suggesting that receptor binding preference is not a sole determinant of these functions. It is clear that HA acquisition of glycans during its evolution in humans can influence HA binding avidity, typically [35,36,37], but not always [22], decreasing binding. As the H3 HA has accumulated glycans, HA specificity has modulated towards branched glycans with extended poly-*N*-acetyl-lactosamine chains capable of bridging two RBSs of single HA trimer to enhance avidity [32,38].

Internalization of cell-associated virions occurs via multiple mechanisms including clathrin/caveolin-dependent and -independent endocytosis or, in the case of filamentous strains, macropinocytosis [39,40,41,42,43]. When the lowering endosomal pH reaches a trigger point that varies with HA strain, HA conformation changes to expose a domain that mediates fusion of viral and cellular membranes, releasing the viral core into the cytosol [44,45,46,47,48,49,50]. Proper acid triggered exposure of the fusion peptide requires cleavage of the HA into two subunits (HA1 and HA2), normally performed by furin or furin-like proteases during or after HA egress through the late secretory pathway [51,52].

Unlike HA, NA is not needed to initiate infection [17], and indeed, inhibiting NA can enhance infectivity [53]. NA plays an essential role in completing the infectious cycle by enabling virion release and preventing HA-mediated aggregation of virions by desialylating HA (and other NA molecules) [54,55,56,57] and possibly virion glycolipids as well. For some strains, HA desialylation is required for HA cleavage by proteases to activate fusion activity [58,59].

## 3. HA/NA Coevolution: NA Perspective

**Figure 1:**
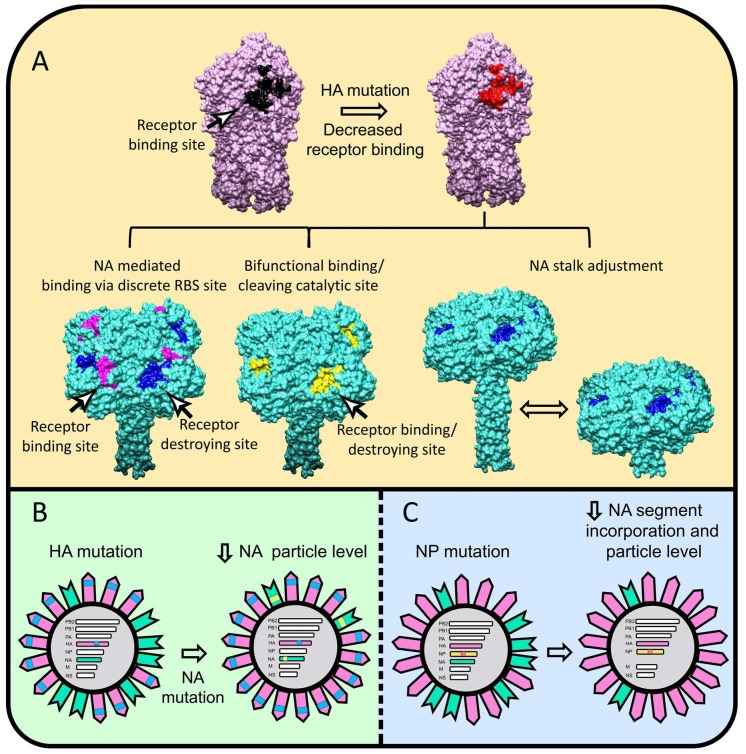
Naturally observed mechanisms to optimize HA–NA stoichiometry. (**A**) The HA H1 (pdb entry 3lzg) is colored pink with wt-RBS in black and mutated RBS with decreased binding in red. The NA N2 is colored turquoise (the model was created by superimposition of pdb entries 2hty and 6crd-containing tetrabrachion stalk) with the receptor destroying/catalytic site in blue, receptor binding site in magenta, and combined receptor binding/destroying site in yellow. (**B**) After the HA (pink) acquires an avidity decreasing mutation (blue), the NA (green) follows with a mutation (bright green) that impairs NA intracellular trafficking and incorporation into virions. (**C**) During host adaptation an NP mutation (red-orange) can decrease NA gene segment incorporation leading to diminished amounts of virion NA to rebalance HA–NA levels. UCSF Chimera 1.13 was used to render the 3-D structures.

As a functional HA antagonist, NA walks a fine line. It must have sufficient activity to release and disaggregate virions, but not so much as to reduce HA-mediated attachment to initiate infection. The earliest and most basic evidence for HA/NA co-evolution is the non-random association of HA and NA subtypes that occur in natural IAV isolates, since it is clear that reassortment is a highly robust process in nature [8,10,11,12] and that co-infections commonly occur in many species with viruses of various subtypes. In one of the first studies to understand this preference, Baum and Paulson reported that N2 NA specificity evolved to match HA specificity during evolution of human N2 viruses from 1957 to 1987 [60]. In the most extreme example of HA–NA cooperation, NA is capable of evolving a sialic acid binding site that functionally replaces the HA binding site (Figure 1A) [32,61,62,63]. This renders IAV into a paramyxovirus-like configuration with a fusion protein (HA = paramyxovirus F protein) and a combined SA receptor/neuraminidase protein (NA = paramyxovirus HN). As with the HN, the NA receptor site is physically separated from the catalytic site. NA is also capable of modifying its active site to simultaneously serve as a sialic receptor for viral attachment, as occurred during the recent human H3N2 viruses [32,64]. Infection of cultured cell by such viruses can be blocked by the NA active site inhibitor oseltamivir, demonstrating functionally that NA replaces HA receptor function, at least in vitro.

NA also evolves subtler alterations that modulate NA specificity or catalytic properties. Before delving into this topic, it is important to recognize that specificity and catalysis are so intimately related that changes to one property can inevitably change the other (this also applies to HA specificity and avidity), given sufficiently discriminatory assays. As the actual in vivo ligands for HA and NA are uncertain and are likely to display enormous heterogeneity and vary considerably based on the precise locale of a given virion, it is wise not to make firm conclusions regarding the functional consequences of measured alterations in NA/HA-glycan interactions.

**Figure 2:**
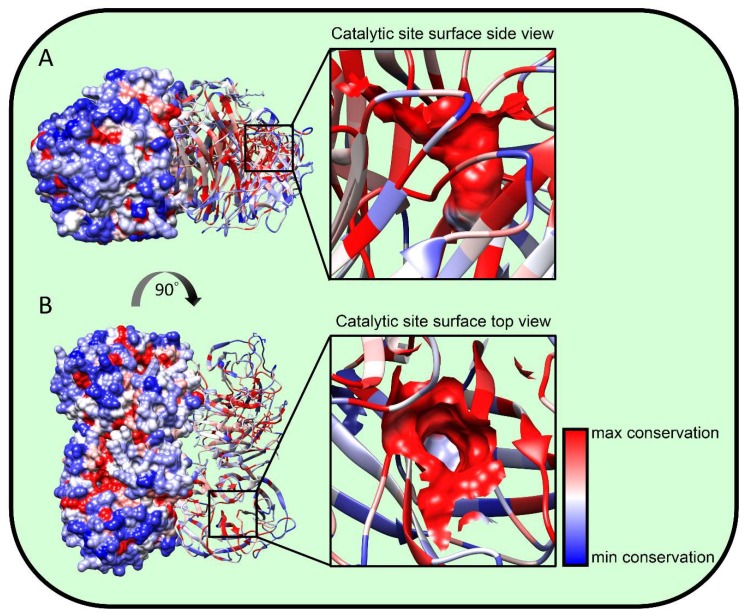
NA structural conservation. We aligned roughly 400 NA amino acid sequences from IAV strains spanning the last 100 years (200 hundred H1N1, 50 H5N1, and 150 H3N2) using MUSCLE software at the fludb website (www.fludb.org). We projected residue conservation onto the N2 NA A/Perth/16/2009 H3N2 (pdb entry 6br5) structure using UCSF Chimera 1.13 software. Blue represents residues with maximal variability while red represents minimal variability. (**A**) Side view showing surface rendering of half the structure with the other half showing ribbon rendering, demonstrating the variability of NA surface residues vs. the conservation of internal residues. (**B**) Top view. The black squares show the catalytic site, which is magnified on the right. The residues forming the catalytic site are surface-rendered; note the high conservation except at the base, which is variable and consistent with its lack of substrate interaction.

The most direct mechanism for NA to adjust its active site properties is to substitute residues in and about the active site. The high conservation of these residues (Figure 2) [55,65] in the absence of NA inhibitor-driven selection indicates that fitness costs limit variation in this region. A less obvious location for functional variation is the fibrous stalk that attaches the globular domain to the membrane. Stalk length governs the height of the globular domain and hence its access to substrates and also its interactions with HA (Figure 1A). Furthermore, it might also allosterically modulate NA activity [66]. Early NA RNA sequencing studies revealed that N1 from viruses isolated between 1933 and 1935 differed in stalk lengths by 11 to 16 residues [67]. Later, it was reported that NA stalk length shortening is frequently observed during adaptation of IAV between avian species [68,69], where it can be associated with increased lethality and transmission [70,71]. Deletions in the NA coding region occurred in H3N2 strains with narrowed receptor specificity, also generating NA inhibitor resistance [72,73]. Molecularly, it is easy to understand how stalk deletions can occur. Indeed, the IAV polymerase may even have evolved a tendency towards bulk nucleotide deletions, as they are a prominent feature of defective interfering (DI) particles, which are present in all virus preparations, and dominate the virion population when the virus is propagated at a high multiplicity of infection (MOI) [74]. The polymerase is also, however, capable of nucleotide insertions by recombination of IAV genes [75] and even by introduction of ribosomal RNA (and likely other host) sequences [76]. Providing one of the first demonstrations of the co-evolution of HA-NA at the molecular level, Mitnaul et al. reported that shortened NA stalk length reduced NA activity to cellular receptors and fitness, with fitness restored by mutations in HA that altered its receptor binding properties [75].

NA function can also be controlled at the level of NA expression to compensate for modified receptor binding and other alterations in HA function. Monoclonal Ab (mAb) selection of HA escape mutations in some instances co-selected NA mutants with single amino acid substitutions that reduce NA virion incorporation, likely due to interference with normal NA assembly or trafficking (Figure 1B) [77,78]. Importantly, some of these NA mutations modified NA sensitivity to clinically used inhibitors and/or modified NA antigenicity. This makes the critical point that NA-HA epistasis muddies interpretation of in vivo evolutionary selection pressures. Thus, it is possible that some of the observed antigenic drift in NA in human IAV evolution is due to selection by HA-specific Abs (and vice versa). A useful guiding philosophy is that extreme caution should be exercised in interpreting the selection pressures responsible for the evolution of genetic alterations.

Adaptation of the mouse A/Puerto Rico/8/1934 (H1N1) strain to guinea pigs revealed another mechanism of controlling NA incorporation into virions, based on a single amino acid substitution in NP that reduced NA mRNA and vRNA expression, reducing NA synthesis and impairing NA gene segment packaging into progeny virions (Figure 1C) [8]. Such semi-infectious (SI) virions lacking one or more gene segments outnumber intact virions in most virus preparations, with the NA segment being most frequently absent [79,80]. SI viruses are relevant in infection since they produce progeny that are easily rescued by recombination, which occurs at high frequency in animal models [8,10,11,12].

## 4. HA/NA Coevolution: HA Perspective

**Figure 3:**
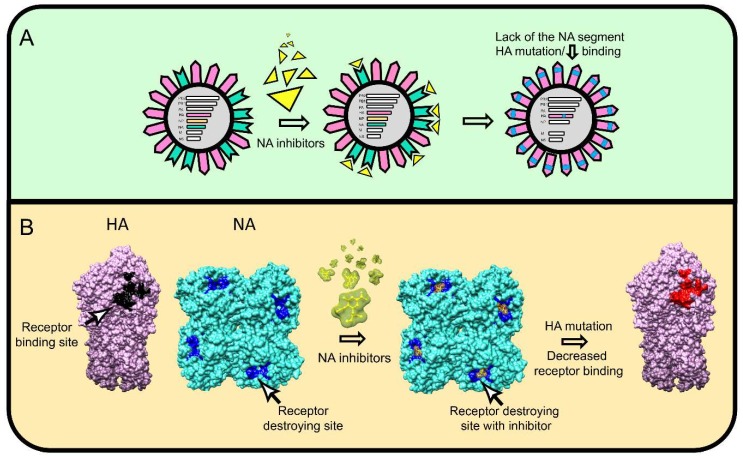
Naturally observed mechanisms of NA–HA epistasis. (**A**) Some oseltamivir-resistant IAV clinical isolates completely lack the NA gene segment and adapt by acquiring mutations that reduce HA receptor avidity. HA is colored pink, NA green, NA inhibitor yellow, HA mutation blue. (**B**) NA inhibitors can select escape mutants with changes exclusively in HA that alter HA receptor binding properties. The H1 HA (pdb entry 3lzg) colored pink with *wt*-RBS black and mutated RBS red, rendered as side view. The N1 NA colored turquoise (pdb entry 3ti6) with or without oseltamivir (yellow-green) bound to receptor destroying site (blue) rendered as top view. UCSF Chimera 1.13 software was used to visualize the molecules.

As a functional NA antagonist, HA also walks a fine line. It must have sufficient avidity to attach virus to cells, but not so much as to reduce NA mediated release from cells or to aggregate viruses that retain terminal SA due to incomplete NA action. In the presence of exogenously supplemented NA, IAV is able to replicate while rapidly acquiring HA substitutions at the tip of RBS (S193R and V205M) that decrease HA–SA binding [81]. Gradually decreasing exogenous NA during virus passage selected an unusual NA-independent strain with HA mutations associated with much weaker SA binding (V135A, S145N located in or near the RBS, and R220K located at the trimer interface). Similarly, the HA substitutions K173E and I260M are selected in oseltamivir resistant H3N2 clinical isolates that, quite remarkably, lack the NA gene segment (Figure 3A) [82].

The widespread clinical use of NA inhibitors provides an opportunity to study how mutations in NA epistatically select for mutations in HA (and potentially other genes) during IAV evolution in humans. Phylogenetic analysis reveals that mutations in HA can favor the emergence of NA inhibitors-resistant variants [83]. Initial in vitro studies found that that NA inhibitor resistant mutations occur in otherwise conserved structural and catalytic NA residues [84,85,86,87]. Surprisingly, however, predominant substitutions, even in some cases with unaltered NA, occurred in multiple HA residues near or in the RBS (N145S, N150S, V90A, L240Q, E116G, T155A, V229I, R229S/I, S165N, K222T, S186F) [84,88,89,90], demonstrating the extensive epitasis between HA and NA.

## 5. Antibody Response to HA and NA

Influenza virions comprise four major structural proteins—M1, HA, NP, and NA—present at a molar ratio, respectively, of ~ 100, 26, 22, and 3 [2]. Under experimental settings, immunization with virions in diverse species (chickens, mice, guinea pigs) results in predominant Ab responses to HA, NA, and NP, as determined by ELISA titers (respectively 55%, 35%, and 10%) [91]. Remarkably, this immunodominance hierarchy is maintained even in lampreys, despite generating Abs using a completely different family of receptors from the Ig receptors used in jawed vertebrates [91].

**Figure 4:**
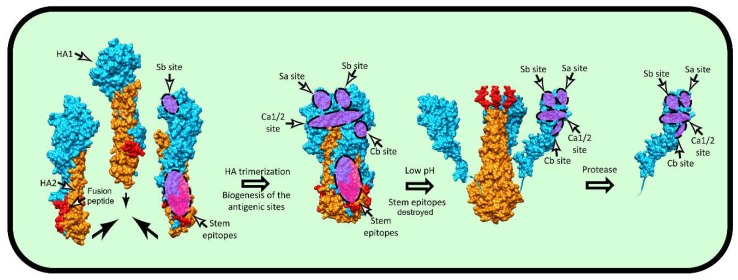
Biogenesis of HA antigenic sites. The HA head antigenic site Sb and HA stem protective epitopes are formed on HA monomers during biogenesis, while for other sites on the HA head, full maturation requires HA trimerization. Contrary to HA stem epitopes, which are destroyed by conformational changes triggered by low pH, HA head antigenic sites are remarkably resistant and remain intact even after proteolytic liberation of the monomeric head domain. The pdb entries 1htm, 1ibn, and 2vir (A/Aichi/2/1968 H3N2) were used to visualize various conformational stages during the fusion process. The HA1 is colored bright blue, HA2 orange, and fusion peptide red. Approximate localization of the canonical antigenic sites and the HA stem epitopes are highlighted by translucent purple with dashed edges. UCSF Chimera 1.13 software was used to visualize the molecules.

The vast majority of anti-HA Abs induced by virus infections, virions, or human vaccines recognize the globular domain, which consists of five canonical antigen sites (Sa, Sb, Ca1, Ca2, and Cb for H1 and A, B, C, D, and E for H3) [92,93,94,95]. A point often missed is that in Caton et al.’s original description of mAb-defined HA antigenicity [96], the full binding of 39 of 89 mAbs is diminished by substitutions in amino acids present in two or more sites. For all but the Sb Ag site, HA trimerization is required to attain full antigenicity [97]. Once formed, neither acid-induced HA head dissociation [98], nor release of the head domain by proteolytic treatment fully disrupts antibody recognition of any of the five major antigenic sites (Figure 4) [97].

**Figure 5:**
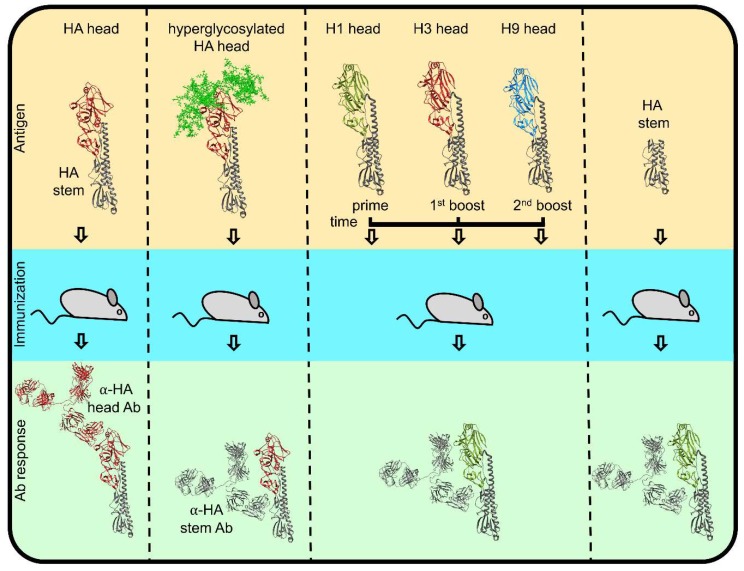
The mechanisms of humoral response modulation towards HA stem domain. If regular HA (top left) is used as an immunogen, the dominant antibody response is directed towards the head domain (dark red). Immunization with hyperglycosylated HA head (complex glycans colored bright green) or sequential immunization with dissimilar head (olive green, dark red, bright blue), identical stem (dim gray) constructs (top middle), or physically separated stem (top right) all result in anti-stem dominated Ab responses.

While the HA globular domain is clearly immunodominant, the much more phylogenetically conserved stem also elicits Abs in humans and experimental animals following infection or vaccination. In initial studies by Russ and colleagues, stem specific polyclonal Abs were found to be specific for denatured HA and exhibited no clear biological activity [99,100,101,102,103,104]. In 1993, however, Okuno et al. [105] identified a stem-specific mAb that neutralized H1 and H2 viruses and later showed that this mAb could provide broad protection in vivo for viruses with group I HA [106]. Although far less abundant than head-specific Abs in human or animal sera [107], Abs specific for the stem domain are induced in all species examined and can attain reasonable, and even high, titers when elicited by native stem vaccines that lack the globular domain [108,109,110] or when the globular domain antigenic sites on intact HA vaccine are blocked by N-linked glycosylation [111]. Repeated exposure to dissimilar HA strains greatly enhances anti-stem responses (Figure 5) [112,113,114], based on the preferential activation of memory vs. naïve B cells. Although exposure of the HA to low pH destroys many stem epitopes recognized by protective Abs [115,116], the biogenesis of several epitopes was shown to be trimerization independent (Figure 4) [117]. While this is potentially good news for producing effective monomeric HA stem immunogens [118], it should be noted that many or even most stem epitopes require HA trimerization to confer full Abs binding [110,117,119,120].

Surprisingly little is known about the immunodominance hierarchy of antigenic sites in the HA globular domain, i.e., the prevalence of Abs and B cells specific for each site. A recent study provided a foundation for mechanistically dissecting B cell and Ab immunodominance in the mouse model. Angeletti et al. [121] created a panel of mAb-selected viruses lacking four of five of the head antigenic sites, enabling measurement of B cells and Abs to individual antigenic sites. This revealed that Abs for the Cb site, as initially reported [122,123], dominate the response early after infection, with anti-Sb Abs attaining dominance by 21 d post infection and continued diversification over time to greatly increase responses to the other antigenic sites. Immunodominance after vaccination with inactivated virus differs, being focused on HA tip region, particularly at Sa and/or Sb sites [121].

The Ab immunodominance hierarchy is important since it is likely to play a central role in determining the selection pressure that drives antigenic drift. Ab selection pressure is the product of the magnitude of Ab response and the functional activity of the Abs against the different sites. Abs that bind the Sa, Sb, Ca1, and Ca2 sites most efficiently block attachment [124,125], with Cb-specific antibodies exhibiting only low potency, consistent with the distal location of the Cb-site from the RBS. Parsing how anti-HA Abs actually work in vivo is a difficult task. Even tip-specific Abs may principally block infection at the level of virus fusion in the endosome activities of at endosomal fusion inhibition [125,126,127], possibly due to a requirement for HA–SA binding to trigger synchronized multi-HA fusion [47] in the late endosome.

Despite the clear anti-virus activity of NA-specific Abs known for 50 years [128] and their well-documented protective effects in humans [129,130,131], NA antigenicity is far less characterized than HA antigenicity. Early studies delineated a number of antigenic sites on N2 HA [132,133,134,135], and recent studies have similarly identified a few epitopes in contemporary N1 NAs [136,137]. The extent to which these epitopes contribute to immunodominant antigenic sites remains to be established.

On a molar basis, NA is the most immunogenic IAV protein in naïve animals [91]. Immunizing with equal molar amounts on HA and NA in humans reveals comparable immunogenicity [138,139]. In IAV-exposed individuals, however, HA immunodominance is enhanced due to immunogen competition, likely based on HA-specific B cells capturing more virions and recruiting more memory Tfh cells, which limit B cell responses [140,141,142].

NA Abs classically function by blocking NA activity against biologically relevant SA-containing substrates that interact with HA. These include cell surface receptors, which otherwise would sequester nascent virions on the cell surface and virion HA itself, which serves as a virion aggregant. NA Abs presumably also function in Fc-based humoral and cellular anti-viral immunity, including complement-based mechanisms, virion phagocytosis, and NK cell mediated effector functions.

With the substantial evidence supporting a protective role for NA antibodies in human immunity to IAV [129,130,131,141,143,144,145], the reader might be surprised to learn that the amount of NA present in human vaccines is not regulated by the FDA, nor publicly reported by the vaccine manufacturers, though it clearly varies enormously between various vaccine formulations. This is difficult to understand, since measuring native NA is far easier than measuring native HA. As an enzyme that requires native structure to maintain enzymatic activity, NA is measured inexpensively and rapidly, simply by its ability to cleave a fluorogenic substrate. In a single afternoon, even a marginal scientist (e.g., an aging PI with waning experimental skills), could measure the NA content of all vaccines used in the world in a given year. In addition, the enzyme-linked lectin assay (ELLA) enables a high throughput determination of functional anti-NA antibodies in serum [146]. This information could then be used to correlate NA vaccine content with the induction of anti-NA Abs and vaccine efficacy.

## 6. Ab-Based NA–HA Cross Talk

The proximity between HA and NA on the limited surface area of the virion raises the possibility that Abs to HA could affect NA function. Note that the opposite event, NA-specific Abs modulating HA function, is much less likely based on the ~10:1 molar dominance of HA. Even at Ab saturation, only a minor fraction of HA will be in proximity to NA-bound Abs, an effect amplified by NA clustering on the virion surface [147,148,149]. After Paniker [150] first reported that anti-HA Abs could inhibit NA activity 50 years ago, Russ et al. [151] showed that this required the physical association of HA with NA on virions, as would be expected. Re-examining this phenomenon using mAbs, mAbs specific for sites at the HA tip (Sa and Sb) were far less efficient at blocking NA activity to fetuin, a 48 kDa soluble glycoprotein substrate, than mAbs that bind to sites lower on HA spike (Ca, Cb) [124].

Extending these findings, mAbs specific for the HA stem were reported to efficiently block NA activity against either fetuin- or SA-containing surfaces, including the cell surface [152,153]. This provided an explanation for the ability of stem-specific Abs to prevent virus release and to thereby reduce viral infectivity [57]. Exploiting the flexible length of the NA stalk, Kosik et al. found that the anti-NA activity of HA-stem specific Abs is inversely proportional to the height of the NA globular domain, which they used to demonstrate the importance of this effect in reducing IAV pathology in mouse lungs and mortality [153]. In as much as the protective capacity of anti-HA stem Abs in mice appears to result from Fc receptor-dependent, innate immune cells [154,155], these findings imply that anti-stem Abs function in vivo by negating the inhibitory effects of virion NA activity on innate immune cell activation. This phenomenon was first reported by Bar-On et al. [156] and is consistent with a requirement for HA engagement of innate immune cell SA residues, as shown by the blocking effects of anti-HA Abs [157,158]. Indeed, the ability of anti-stem mAbs to activate NK reporter cells was proportional to their steric inhibition of NA activity [153]. As stem-specific Abs are possibly the basis for universal vaccination, it is critical to understand how they mediate protection.

These findings underscore the complexity of humoral immunity to IAV and the difficulties in assessing the contribution of various Ab-based effector mechanisms to protection, as well as to the evolution of HA and NA. Pragmatically, there is a potential danger in vaccination that leads to a stem response whose protection is abrogated by a head response sufficient to block innate immune cell activation but insufficient to protect via standard virus neutralization.

## 7. Future Directions

It is clear that HA and NA intimately function as a unit to enhance virus transmission and consequently co-evolve to optimize their overall fitness. Without doubt the segmentation of IAV facilitates this co-evolution as it enhances the ability of a virus population to sample permutations of HA and NA genes through co-infection, which appears to be a common event in vivo. Indeed, the abundance of semi-infectious virions lacking the NA gene [80] ensures that HA genes will widely sample NA genes to generate fully infectious viruses capable of inter-host transmission, which is severely bottlenecked in humans [3]. The intertwined relationship between HA and NA is reflected in the function of anti-HA Abs, which sterically block NA access to biological substrates in a manner dependent on the location of their epitopes on the HA spike and the length of the NA stalk.

These findings raise numerous questions for future studies, including:

(1) How does the geometric distribution of HA and NA on the virion effect their functions? Why is NA clustered on virions? Is the extent of clustering variable between IAV strains? How does the relationship between NA and HA alter in filamentous vs. spherical virions? How does the stoichiometry of virion HA and NA influence viral function? What mechanisms does the virus use to control HA–NA virion content?
(2) How do Ab-heavy chains influence the functional activities of anti-HA Abs on NA activity, particularly the larger oligomeric structure of IgM and IgG? Further, what is the functional impact of the effect of Ab binding molecules such as complement?
(3) What is it exactly about HA and NA that enables their rapid antigenic evolution, while analogous proteins on other viruses evolve much more slowly? Is it the freedom for independent mutations afforded by a segmented genome? Is it some inherent resistance to in vivo Ab-mediated neutralization in humans that is not apparent in animal models? Is it something about the immunodominance hierarchy in individuals that enables sequential escape across a population?

Humanity has been studying IAV for nearly 90 years. While great strides have been made in understanding many aspects of viral replication, immunity, and pathogenesis, essential information needed to improve vaccination remains elusive. Despite its diminutive genome, IAV is a formidable foe. Deciphering its secrets will take a concerted effort and requires a reappraisal of what we think we know. The field should welcome with open arms young minds and those from different backgrounds who will be less burdened by preconceived opinions and more likely to take creative approaches to answering known questions and posing new ones.
